# Arrhythmogenic late Ca^2+^ sparks in failing heart cells and their control by action potential configuration

**DOI:** 10.1073/pnas.1918649117

**Published:** 2020-01-22

**Authors:** Ewan D. Fowler, Nan Wang, Melanie Hezzell, Guillaume Chanoit, Jules C. Hancox, Mark B. Cannell

**Affiliations:** ^a^School of Physiology, Pharmacology and Neuroscience, Faculty of Biomedical Sciences, University of Bristol, University Walk, BS8 1TD Bristol, United Kingdom;; ^b^Bristol Veterinary School, University of Bristol, BS40 5DU Bristol, United Kingdom

**Keywords:** heart, arrhythmia, cardiac myocytes, action potential, Ca^2+^ sparks

## Abstract

Sudden death in heart failure patients is a major clinical problem worldwide, but it is unclear how arrhythmogenic early afterdepolarizations (EADs) are triggered in failing heart cells. To examine EAD initiation, high-sensitivity intracellular Ca^2+^ measurements were combined with action potential voltage clamp techniques in a physiologically relevant heart failure model. In failing cells, the loss of Ca^2+^ release synchrony at the start of the action potential leads to an increase in number of microscopic intracellular Ca^2+^ release events (“late” Ca^2+^ sparks) during phase 2–3 of the action potential. These late Ca^2+^ sparks prolong the Ca^2+^ transient that activates contraction and can trigger propagating microscopic Ca^2+^ ripples, larger macroscopic Ca^2+^ waves, and EADs. Modification of the action potential to include steps to different potentials revealed the amount of current generated by these late Ca^2+^ sparks and their (subsequent) spatiotemporal summation into Ca^2+^ ripples/waves. Comparison of this current to the net current that causes action potential repolarization shows that late Ca^2+^ sparks provide a mechanism for EAD initiation. Computer simulations confirmed that this forms the basis of a strong oscillatory positive feedback system that can act in parallel with other purely voltage-dependent ionic mechanisms for EAD initiation. In failing heart cells, restoration of the action potential to a nonfailing phase 1 configuration improved the synchrony of excitation–contraction coupling, increased Ca^2+^ transient amplitude, and suppressed late Ca^2+^ sparks. Therapeutic control of late Ca^2+^ spark activity may provide an additional approach for treating heart failure and reduce the risk for sudden cardiac death.

## Significance

Sudden cardiac death in heart failure is a major unsolved clinical problem that is linked to the development of a spontaneous arrhythmia. Early afterdepolarizations (EADs) are an arrhythmogenic mechanism, but the cellular trigger for EADs in heart failure is unclear. We show that the reduction in synchronous Ca^2+^ release early in the action potential (AP) of failing cardiac myocytes promotes the appearance of late Ca^2+^ sparks which can propagate, forming Ca^2+^ ripples and waves. These, in turn, produce an inward sodium–calcium exchange current which opposes AP repolarization. Restoration of AP phase 1 repolarization improved Ca^2+^ release synchrony and reduced late Ca^2+^ spark rate, suggesting a different approach to reducing the risk of sudden death in heart failure.

Worldwide, ∼26 million people suffer from heart failure (HF) (1). More than 50% of HF patients die suddenly, and this sudden cardiac death is most likely due to the spontaneous emergence of arrhythmias (2, 3). At the cellular level, two identified initiators of cardiac arrhythmias are delayed afterdepolarizations (DADs) and early afterdepolarizations (EADs) (4). DADs occur in the resting period between heart beats (diastole) and are due to spontaneous Ca^2+^ release from the sarcoplasmic reticulum (SR) in the form of Ca^2+^ sparks (5), which summate to form propagating Ca^2+^waves (6, 7). These waves cause a depolarizing inward current via the Na^+^/Ca^2+^ exchange (NCX) mechanism (6, 8, 9). In contrast to DADs, EADs are less well understood and occur during the repolarization phase of the cardiac action potential (AP) where several ionic currents interact to control repolarization (10). EADs can be produced by reactivation of ionic currents during AP repolarization when the potassium currents forming the “repolarization reserve” (11, 12) are insufficient to maintain the repolarization trajectory of the AP, although why this should occur spontaneously within a steady train of APs is uncertain (13). Spontaneous Ca^2+^ waves have also been implicated in EAD generation (9), but it is unclear how such waves might be initiated when the SR should be depleted and/or refractory after SR Ca^2+^ release is triggered by the upstroke of the AP (14).

We recently showed that “late” Ca^2+^ sparks (LCS) can occur during the decay of the Ca^2+^ transient in normal rabbit cardiac myocytes (15). Here, we report that stochastic LCS and low-amplitude “Ca^2+^ ripples” are promoted in HF and can generate sufficient NCX current (I_NCX_) to trigger EADs. In addition, normalizing the early AP waveform in HF promotes a faster and larger SR Ca^2+^ release (which should improve contractility) and at the same time decreases arrhythmogenic LCS activity.

## Results

**Fig. 1.**
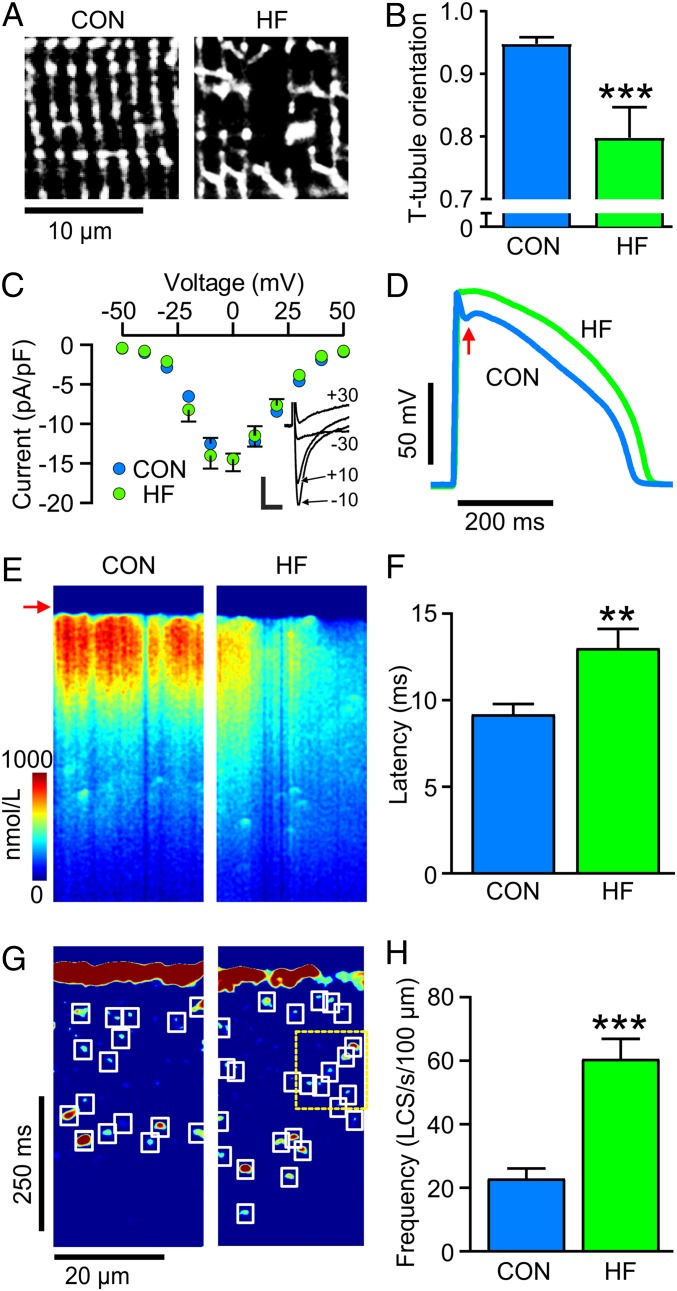
Changes in structure, AP time course, t-tubule morphology, and increased LCS in HF. (*A*) di-8-ANEPPS staining shows reduced tubule regularity and (*B*) reorientation of t-tubules in HF cells compared to control (CON). *n*/*N* = 9/4 CON 12/5 HF. (*C*) I_Ca_ density was unchanged in HF. *n*/*N* = 20/4 CON 10/3 HF Representative currents at the indicated test potentials (mV) are shown (*Inset*). Error bars not shown where error is less than the size of symbols. (Scale bars: 5 pA/pF, 10 ms.) (*D*) APs in HF myocytes had less AP phase 1 repolarization (arrow). (*E*) Ca^2+^ transients in CON myocytes showed synchronous Ca^2+^ release, while HF showed less uniform Ca^2+^ release and (*F*) an increase in latency from electrical stimulation to detectable Ca^2+^ increase. (*G*) Image processing reveals increased LCS frequency in HF with some Ca^2+^ ripples (yellow dashed box). (*H*) LCS frequency was greater in HF cells than in CON. *n*/*N* = 16/6 CON; 14/5 HF. **P < 0.01, ***P < 0.001, unpaired *t* tests.

LCS occur in a wide variety of species (*SI Appendix*, Fig. S1), but, to study repolarization mechanisms, an AP similar to human is desirable, and we therefore used an established rabbit model of chronic myocardial infarction that causes HF. When left ventricular ejection fraction had fallen to 44 ± 3% (*SI Appendix*, Fig. S2 and Table S1), indicative of moderate to severe HF, ventricular myocytes exhibited t-tubule remodeling (Fig. 1 *A* and *B*) similar to that seen in most models of HF (16). This “t-tubule disease” (17) decreases the efficiency of excitation–contraction coupling and the rate of rise of the Ca^2+^ transient (*SI Appendix*, Fig. S3) (18, 19). Although there was no change in I_Ca_ density (Fig. 1*C*) in this HF model, the AP lost phase 1 repolarization (red arrow, Fig. 1*D*) and became prolonged (*SI Appendix*, Fig. S3). All of these changes are also seen in human HF (17, 20). The loss of AP phase 1 contributes to the reduced SR Ca^2+^ release rate (Fig. 1*E* and *SI Appendix*, Fig. S3) and increased SR release latency (Fig. 1*F*) (16, 18) because this phase of the AP affects the magnitude and time course of I_Ca_ which triggers SR Ca^2+^ release (21, 22).

The reduction in synchronous Ca^2+^ release in HF, seen as a less uniform increase in Ca^2+^ after the AP upstroke (Fig. 1*E*, arrow), was associated with an increase in the number of LCS (Fig. 1 *G* and *H*). This ∼3-fold increase in LCS frequency can be explained by an increase in the number of Ca^2+^ release sites that were not triggered earlier during the AP (15, 22). The increased availability of Ca^2+^ release sites also promotes sequential activation of LCS, resulting in Ca^2+^ ripples (15), an example of which can be seen within the yellow dashed box in Fig. 1*G*.

### Linkage between LCS and Arrhythmogenesis.

**Fig. 2.**
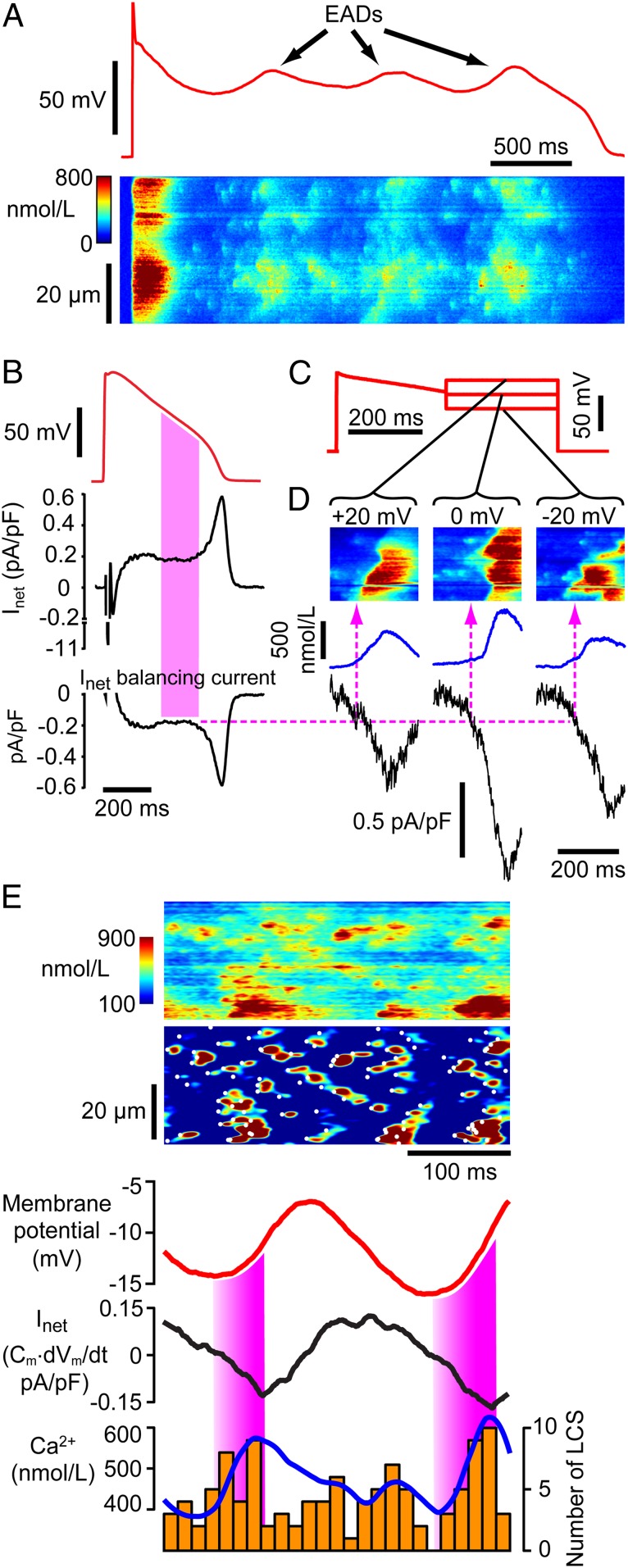
LCS can trigger EADs. (*A*) EADs evoked by pacing-pause protocol. During repolarization, an initial V_m_ inflection at ∼ −20 mV generates EADs (*Top*), and numerous LCS create oscillatory Ca^2+^ release (*Bottom*). (*B*) Calculation of I_net_ for a typical HF AP. The filled region indicates where the greatest number of LCS occur (*A* and Fig. 4*E*) and corresponds to an I_net_ of ∼0.18 pA/pF. Any balancing current that counteracts I_net_ (i.e., −I_net_, *Bottom*) will stop repolarization (*SI Appendix*, Fig. S5). (*C*) Failing AP with voltage-clamp steps used to probe Ca^2+^ and currents underlying EADs. (*D*) During the AP steps, LCS-triggered Ca^2+^ waves (*Top*) and inward currents (black trace) appear. Dashed line shows the inward current that balances I_net_ in *B* and could therefore stop repolarization. This current occurs when LCS are about to initiate Ca^2+^ waves. (*E*) Example of apparently chaotic Ca^2+^ during EADs in an HF cell (*Top*). Image processing reveals many LCS and Ca^2+^ ripples during V_m_ oscillations (red trace). The filled regions show where the depolarizing current (I_net_) is increasing, and this corresponds to increasing Ca^2+^ (blue) and LCS activity (orange bars).

LCS activity depends on SR Ca^2+^ content (15) which generally increases with AP frequency (10, 23). A pacing-pause protocol increases SR Ca^2+^ and may provoke EADs (24). With this protocol, LCS activity increased, and EADs appeared (Fig. 2*A*). The high rate of LCS production (and appearance of Ca^2+^ ripples; see *SI Appendix*, Fig. S4) in these conditions clearly opposed the normal decline of Ca^2+^ during the Ca^2+^ transient.

In order to generate an EAD, the net cellular repolarizing current (I_net_) and/or repolarization reserve (11, 12) must be decreased and/or counteracted by depolarizing membrane current. While I_net_ is the sum of all inward and outward currents, the rate of change of membrane potential (dV_m_/dt) is directly proportional to I_net_ because I_net_ = −C_m_·dV_m_/dt (where C_m_ is membrane capacitance). Applying this equation allows us to determine how much current is responsible for the repolarization phase of the AP, and Fig. 2*B* illustrates the magnitude and time course of I_net._. In this exemplar, I_net_ was only ∼0.18 pA/pF during the late AP repolarization phase, and the average I_net_ during AP repolarization was 0.26 ± 0.08 pA/pF (mean ± SD *n*/*N* = 15/4), showing that a small change in membrane currents will affect repolarization trajectory. It follows that any additional inward current of similar amplitude to I_net_ could stop AP repolarization (*SI Appendix*, Fig. S5) and thereby initiate an EAD.

An increase in intracellular Ca^2+^ moves the electrochemical gradient for NCX Ca^2+^ transport outward, causing an inward shift of I_NCX_ (25) which can be up to ∼1 pA/pF in magnitude (26). To examine whether the observed LCS activity could produce sufficient I_NCX_ to oppose I_net_, we voltage-clamped cells with a failing AP which included steps to different holding potentials around the times (and V_m_) where EADs occur (Fig. 2*C*). During these V_m_ steps, many LCS occurred which triggered Ca^2+^ ripples and Ca^2+^ waves. The average Ca^2+^ (blue trace) and evoked inward current (black trace) are shown in Fig. 2 *D*, *Lower*. This inward current had a linear relationship to mean Ca^2+^ with a slope of −1.5 pA/pF/µmol/L (*SI Appendix*, Fig. S6), compatible with the expected Ca^2+^ dependence of I_NCX_ (26). The critical inward current (from Fig. 2*B*) that will balance I_net_ and stop repolarization is indicated by the dotted line which, after projection onto the Ca^2+^ records, reveals the Ca^2+^ changes associated with this critical current density. It is notable that this current density is developed just before the onset of Ca^2+^ waves which appear as chevron patterns in line scans (5, 7), showing that LCS and/or Ca^2+^ ripples can be the initiating events for EADs.

Fig. 2*E* shows that multiple Ca^2+^ ripples, rather than Ca^2+^ waves, can also give rise to EADs in current-clamped cells. In this exemplar, the line scan Ca^2+^image (upper panel) is rather chaotic with no obvious Ca^2+^ waves being present. However, after image processing, numerous LCS events are seen, some of which form propagating Ca^2+^ ripples (cf. *SI Appendix*, Fig. S4). V_m_ and I_net_ can be linked to Ca^2+^ by two mechanisms: (1) Either Ca^2+^ activates inward I_NCX_ producing an inward shift of I_net_ and depolarization of V_m_ or (2) V_m_ activates L-type Ca^2+^ channels (LTCCs) which then trigger SR Ca^2+^ release. As shown by the colored bars in Fig. 2*E*, the rise in Ca^2+^ occurred in synchrony with inward I_net_ (peak value ∼ 0.15 pA/pF) which also supports the idea that the initiation of EADs can result from the summation of LCS and Ca^2+^ ripples which activate inward I_NCX_.

### Model Analysis of the Role of I_Ca_ and I_NCX_ in EADs.

**Fig. 3.**
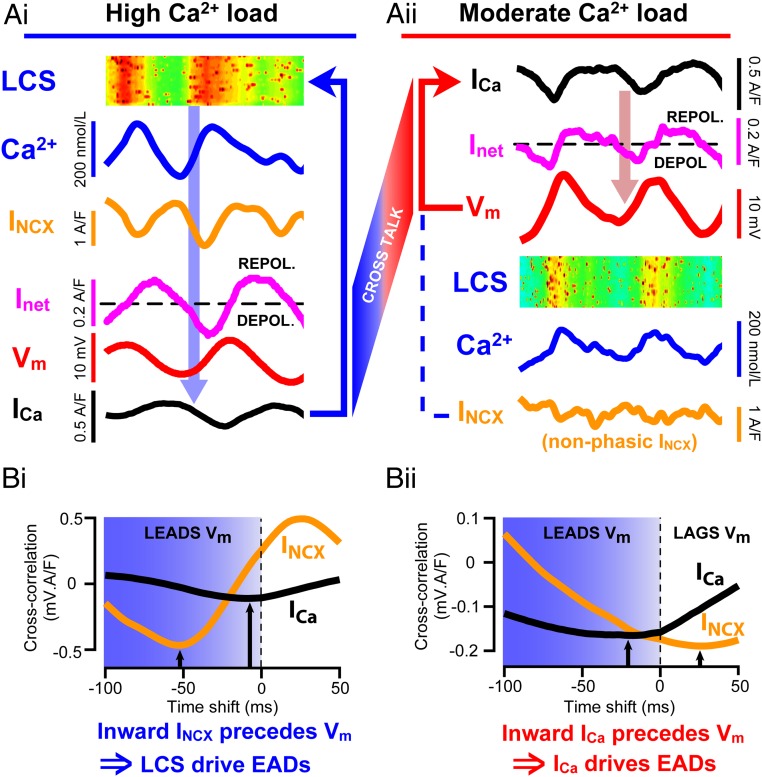
Computer simulations of experimental traces using a model that generates LCS (29). Panels are ordered to indicate the flow of causation. (*Ai*) Increased LCS frequency is in phase with inward I_NCX_ and dV_m_/dt, which precedes V_m_ and I_Ca_ forming an oscillatory feedback pathway as shown by the arrows. (*Aii*) When Ca^2+^ load is reduced, V_m_ oscillates because I_Ca_ (and possibly I_Na_) supply the inward current to increase dV_m_/dt and cause the EAD, as shown. In this case, LCS (and Ca^2+^) are the consequence of the reactivation of I_Ca_. Note that in this simulation, I_NCX_ bears no obvious relation to dV_m_/dt (unlike the results shown in *Ai*). Cross-talk between the oscillators shown in *Ai* and *Aii* can occur because they contain common mechanisms. (*B*) Cross-correlograms between V_m_ and both I_Ca_ and I_NCX_ from the data shown in part A. The negative peaks in the cross-correlogram (black arrows) reveal both the strength of association (amplitude) and temporal relationship (offset from V_m_) between V_m_ and I_Ca_ or I_NC_X (see *SI Appendix*). (*Bi*) When Ca^2+^ load was high, inward I_NCX_ preceded V_m_ oscillations and was more strongly correlated with V_m_ than I_Ca_. (*Bii*) Under reduced Ca^2+^ load, I_NCX_ lags behind V_m_ oscillations. I_Ca_ reactivation preceded V_m_ and so has the required temporal relationship to explain EAD initiation, although some inward I_NCX_ may still contribute (13).

The importance of I_NCX_ for EAD generation has also been demonstrated in murine cells by heterozygous NCX knockdown and in failing rabbit ventricle by acute NCX blockade (27, 28). To further test the idea that sufficient I_NCX_ can be generated by LCS to trigger an oscillatory EAD, we modified a new computer model of spatially distributed Ca^2+^ signaling (29) to calculate membrane currents that should occur in response to experimental V_m_ and Ca^2+^ signals (Fig. 3*A*). This approach breaks the intrinsic feedback loop(s) that otherwise make experimental dissection of causation problematic. Utilizing the recorded Ca^2+^ and V_m_ data shown in Fig. 2 as exemplar controlling variables, the model exhibited two types of behavior: when Ca^2+^ and LCS activity was high (Fig. 3*Ai*), I_NCX_ was in phase with dV/dt and of sufficient magnitude to explain the start of the V_m_ oscillations (cf. Fig. 2 *D* and *E*). However, when SR Ca^2+^ release was slightly reduced, changes in I_NCX_ were much smaller because the change in V_m_ opposed the change in electrochemical gradient for Ca^2+^ extrusion by NCX (Fig. 3*Aii*). The relative roles of I_NCX_ and I_Ca_ in initiating the EAD can be examined by cross-correlation with V_m_. The magnitude of the negative peak in the cross-correlogram shows the strength of the linkage between these inward currents and V_m_, while the time delay associated with the peak in the correlogram indicates whether they are the consequence of a change in V_m_ (by lagging behind V_m_) or a potential cause (by leading the change in V_m_). When the Ca load was high, inward I_NCX_ clearly preceded the change in V_m_ (Fig. 3*Bi*), consistent with the idea that the EADs were triggered by LCS activity and the consequent change in I_NCX._ In comparison, I_Ca_ was both more weakly correlated and almost in phase with V_m_. When SR load was decreased, the reduction in LCS activity led to a smaller inward I_NCX_ being less well correlated with V_m_ and with inappropriate phase to explain EAD initiation (Fig. 3*Bii*). However, I_Ca_ became more strongly correlated with V_m_ (due to a decrease in Ca^2+^-dependent LTCC inactivation (10)) and had appropriate timing to explain EAD initiation. Thus, while both mechanisms for EAD generation were present in the model, an increase in Ca^2+^ release caused the dominant inward current trigger for EAD generation to shift from I_Ca_ reactivation to LCS-evoked I_NCX_.

### Improving Ca^2+^ Signaling and Suppressing LCS in HF Cells.

**Fig. 4.**
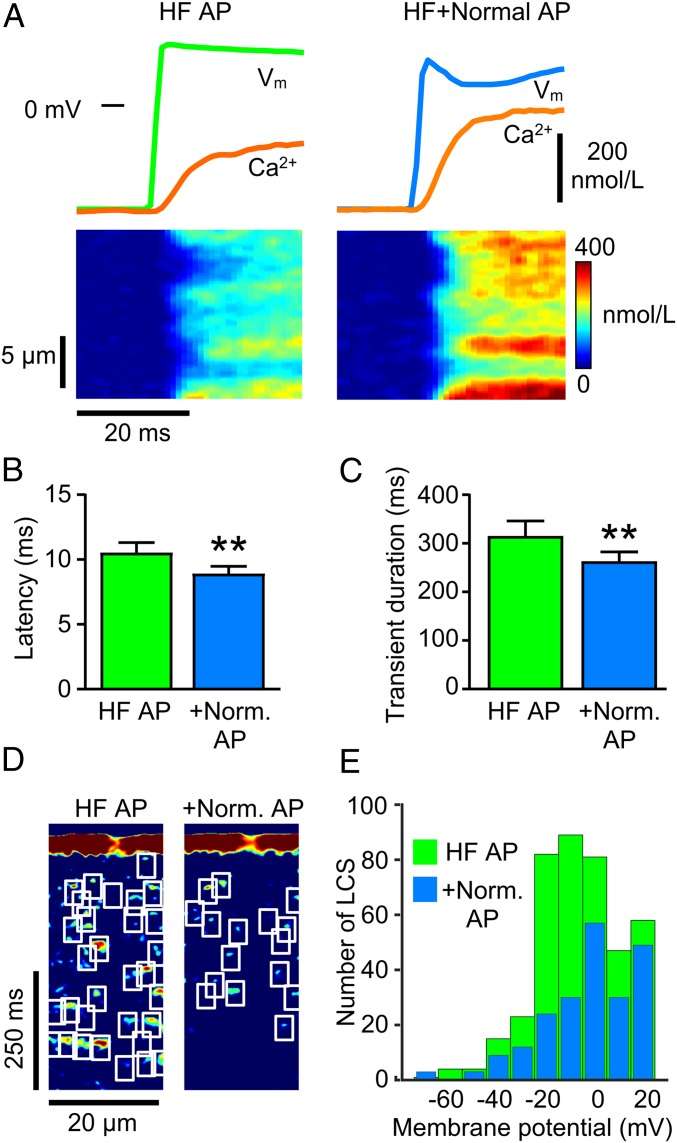
Applying a normal AP to HF cells improves excitation–contraction coupling and reduces LCS frequency. (*A*) Ca^2+^ transient upstroke velocity, amplitude (orange), and Ca^2+^ release synchrony all increase in an HF cell (*Left*) when voltage-clamped with a normal AP (*Right*). (*Lower*) Improved synchrony in Ca^2+^ release. (*B*) Mean latency for Ca^2+^ release and (*C*) Ca^2+^ transient duration were reduced in HF cells clamped with a normal AP. (*D*) Processed Ca^2+^ images show fewer LCS in HF after applying normal AP. (*E*) Number of LCS detected in HF with failing AP waveform (HF AP, 404 LCS) and with a normal AP (+Norm AP, 217 LCS) from 13 HF cells binned by V_m_ during the AP repolarization. **P < 0.01, paired *t* test *n*/*N* = 13/5.

Fig. 4*A* shows that a normal AP applied to HF cells increased the rate of rise and amplitude of the Ca^2+^ transient, and this was associated with an increase in Ca^2+^ release synchrony. On average, the normal AP increased the rate of rise of the Ca^2+^ transient from 30 ± 8 nmol/L/ms to 40 ± 9 nmol/L/ms (*P* < 0.01, paired *t* test), while the time to peak decreased from 75 ± 9 ms to 65 ± 9 ms (*P* < 0.01, paired *t* test). This improvement in excitation–contraction coupling efficiency was also reflected by a decrease in the latency for Ca^2+^ release (Fig. 4*B*). With the improvement in synchrony of early release, the duration of the Ca^2+^ transient became shorter (Fig. 4*C*) due to an ∼50% decrease in LCS frequency (Fig. 4 *D* and *E*). These results confirm that at least a part of the nonuniform and delayed SR Ca^2+^ release seen in the HF model is due to AP phase 1 effects on I_Ca_.

It may not be possible to completely restore the maximum rate of rise, time to peak Ca^2+^, and Ca^2+^ transient duration to CON values with phase 1 modification alone because (1) the t-tubule disruption associated with HF would still be present and (2) there is reduced SERCA2a expression in HF which decreases SR Ca^2+^ uptake rate (30). Nevertheless, it is apparent that AP restoration can improve the defective Ca^2+^ signaling seen in HF and, importantly, reduce the LCS activity that can trigger EADs.

## Discussion

**Fig. 5.**
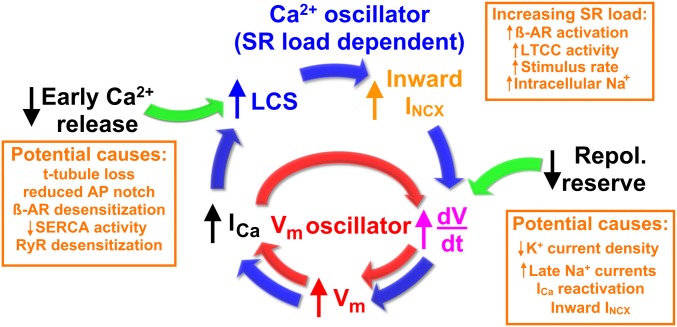
Flow diagram for two interacting positive-feedback mechanisms that can drive V_m_ and Ca^2+^ oscillations during EADs. The red inner cycle represents the well-established V_m_ oscillator, wherein an increase in dV_m_/dt (caused by a relative increase in depolarizing currents compared to repolarizing currents, e.g., text in orange box at *Lower Right*) leads to voltage-dependent reactivation of I_Ca_, which in turn causes further depolarization. The blue outer cycle represents the stochastic LCS-mediated Ca^2+^ oscillator mechanism for EAD initiation. Reduced early Ca^2+^ release and/or increased SR load increases LCS production, and the resulting increase in inward I_NCX_ tends to depolarize the membrane which then feeds onto I_Ca_ to trigger additional LCS. Green arrows indicate possible factors contributing to EAD initiation. β-AR, beta-adrenoreceptor.

Reactivation of I_Ca_ and I_Na_ to overcome the repolarization reserve and initiate EADs has been widely considered to be the primary mechanism for EAD generation (illustrated by the inner feedback loop shown in Fig. 5), e.g., refs. 11, 31, and 32. Indeed, when SR Ca^2+^ release was inhibited by blocking LTCCs, it was still possible to evoke EADs by a simulated I_Ca_ (33). Here we show that LCS-activated inward NCX current can initiate EADs and, we suggest, act as a coupled oscillator to further increase the risk for development of multiple EADs (outer loop in Fig. 5). This Ca^2+^ oscillator arises from two connected mechanisms: (1) Diffusion of Ca^2+^ from an initiating LCS may trigger additional LCS due to the gain inherent in Ca^2+^-induced Ca^2+^ release (CICR). This SR load-dependent process manifests as propagating Ca^2+^ ripples and, if enough LCS sites are available, more synchronous and larger Ca^2+^ waves. (2) LCS will increase inward NCX current to depolarize V_m_ (34) while V_m_ couples back onto LCS activity via the V_m_ dependence of LTCC gating (which may explain the V_m_ dependence of LCS frequency shown in Fig. 4*E*). The stochastic nature of LCS provides an explanation for the sudden appearance of arrhythmogenic EADs, and, once started, an EAD will promote additional LCS and EADs due to the increased SR Ca^2+^ load arising from I_Ca_ reactivation.

The computer model clearly showed that both the electrical (V_m_) and Ca^2+^ oscillators can couple and synergize (Fig. 4*A*). While the phase relationship between LCS-dependent NCX currents and dV_m_/dt directly supports the idea that I_NCX_ can initiate Ca^2+^ dominant oscillations, I_Ca_ reactivation is also important (35) because late LTCC openings are a potent trigger for LCS (15). However, when Ca^2+^ levels are lower, and Ca^2+^ ripples and waves cannot form, EADs can still arise from instability in the repolarization process due to recruitment of noninactivated inward currents in the presence of insufficient repolarization reserve (11). In the latter case, smaller numbers of LCS may still play a lesser role in EAD initiation, and this is reminiscent of the role played by diastolic Ca^2+^ sparks in cardiac pacemaking by sino-atrial node cells (36).

### Ca^2+^ Ripples, Ca^2+^ Waves, and V_m_ Oscillator Coupling.

Although Ca^2+^ waves (Fig. 2*D*) have been implicated in EAD genesis (37), it is notable that the fluctuations in Ca^2+^ shown in Fig. 2 *A* and *E* are not typical Ca^2+^ waves (as seen in Fig. 2*D*) (6, 7); instead, the Ca^2+^ fluctuations came from low-amplitude Ca^2+^ ripples. In such cases, the local propagation of Ca^2+^ release (short-range Ca^2+^ ripples) may not be able to initiate cell-wide Ca^2+^ waves because the effective amplification of Ca^2+^ release by CICR is insufficient for full Ca^2+^ wave support. This lack of sufficient amplification could be explained by the stochastic nature of LCS, coupled with local SR refractoriness (15, 38), decreasing the recruitment of adjacent LCS sites. Nevertheless, some synchronization in Ca^2+^ ripple initiation must occur for average Ca^2+^ to oscillate. The mathematical basis for the emergence of macroscopic Ca^2+^ oscillations from large numbers of independent LCS oscillators is beyond the scope of this study, but Kuramoto model analysis has shown that macroscopic oscillations can develop even when coupling across individual oscillators is weak (39). The relative importance of the Ca^2+^ and V_m_ oscillators in EAD generation will be variable because they depend on many factors such as the actual V_m_ trajectory, K^+^ current availability, the level of SR Ca^2+^ loading in the cell, and I_Ca_ availability, as well as RYR2 Ca^2+^ sensitivity, as illustrated in Fig. 5. In connection with this point, increased LTCC activity and SR load associated with β-adrenergic stimulation (10) would almost certainly increase the risk for LCS-stimulated EADs.

It should be noted that the LCS activity seen in our confocal line scans reflects only a small fraction of the actual number of LCS occurring in the entire cell; the confocal line scan surveys ∼2% of the cell volume, so ∼1 LCS/ms (Fig. 2*E*) would correspond to ∼50 LCS/ms cell-wide, and this much larger number underlies the measured NCX current that can initiate the EAD. This estimate is in reasonable agreement with the computer model used here which predicts that 107 LCS/ms may generate 0.26 pA/pF NCX current (29).

Since LTCC activity is common to both the V_m_ and Ca^2+^ oscillators, the idea that LTCC gating modification could be a therapeutic target for EAD prevention (33) becomes even more attractive. In addition, if modifying the late component of I_Ca_ is able to reduce net Ca^2+^ influx into the cell, SR Ca^2+^ content might be reduced (40), and this would also inhibit LCS activity (15). The improvement in Ca^2+^ signaling produced by applying a normal AP to HF myocytes is remarkable. This suggests that new therapies should be developed with the aim of improving early Ca^2+^ release by restoring phase 1 repolarization and/or restoring t-tubule regularity. This would reduce LCS frequency and thereby reduce the risk for potentially lethal LCS-triggered arrhythmias as well as mitigate the defective excitation–contraction coupling seen in HF (41).

## Materials and Methods

More extensive details are available in *SI Appendix*. Briefly, all experiments were performed in accordance with the UK Home Office Animals (Scientific Procedures) Act 1986 and institutional approval by the University of Bristol ethics committee. We used an established coronary artery ligation model that leads to heart failure in adult New Zealand White rabbits (3–3.5 kg) which were daily monitored for health status. The target endpoint for HF was an ejection fraction of 40% (as measured by echocardiography *SI Appendix*, Fig. S2). Some rabbits did not quite reach this endpoint but presented other indicators of heart failure including dilated left ventricle and lung congestion (*SI Appendix*, Table S1). This model, due to repolarizing current behavior, can be used to gain insight into repolarization reserve with human relevance (42). It was not possible to wait for arrhythmias to start in this model (as they would be fatal), but EADs can be provoked in vitro by suitable interventions. Rather than use pharmacological manipulation to provoke EADs, we chose a pacing-pause method to minimize other possible perturbations of cellular function.

### Cardiac Myocyte Isolation.

Left ventricular epicardial myocytes were obtained from rabbit hearts after full anesthesia (50 mg/kg sodium pentobarbital i.v.) and euthanasia. Enzymatic dissociation was carried out using 1 mg/mL collagenase I (Worthington), 0.05 mg/mL protease (type XIV Sigma), and 0.1 mmol/L Ca^2+^, as described previously (22). Guinea pig, rat, mouse, and zebrafish myocytes were isolated using similar methods to those described previously (22, 43, 44), and methods for zebrafish myocyte isolation are given in *SI Appendix*.

### Electrophysiology.

Electrophysiology experiments were performed in a modified Tyrode’s solution (containing, in mmol/L: 133 NaCl, 5 KCl, 1 NaH_2_PO_4_, 10 4-(2-hydroxyethyl)-1-piperazineethanesulfonic acid (HEPES), 10 glucose, 1.8 CaCl_2_, 1 MgCl_2_, pH 7.4 with NaOH) at 36 ± 1 °C. Patch pipettes were pulled from borosilicate glass using a P80 micropipette puller (Sutter Instruments). Pipettes were filled with an intracellular solution containing, in mmol/L: 120 aspartic acid, 20 KCl, 10 HEPES, 10 NaCl, 5 glucose, 5 Mg.ATP, 0.05 Fluo-4 pentapotassium salt, with KOH added to adjust to pH 7.2. Tip resistance was typically 1.6–2.0 MΩ when filled with this solution. Membrane potential and currents were recorded using an Axopatch 1D amplifier (Molecular Devices), Power1401 digitizer (Cambridge Electronic Design), and Signal data acquisition software (version 6.04, Cambridge Electronic Design). Cell membrane capacitance was measured by step depolarizations to −75 mV from a holding potential of −80 mV for 25 ms. Series resistance was compensated by ∼70%. Liquid junction potential (10 mV) was subtracted from recordings.

### Confocal Imaging.

Ca^2+^ sparks and transients were recorded in line scan mode (45) from the fluo-4 loaded cells using an inverted confocal microscope (LSM 880, Zeiss) with a 1.4 NA 63× oil immersion lens. Excitation light was provided by a 488-nm argon laser, and fluorescence emission was collected at 492–600 nm. Ca^2+^ line scans were recorded with the pinhole set to <2 Airy units, at a pixel size of 0.1–0.2 μm/pixel, and with a scan speed of 1 ms per line. GaAsP photodetectors were used to increase the sensitivity of Ca^2+^ spark detection. The t-tubule system was imaged by labeling the sarcolemma with di-8-ANEPPS from a stock 1 mmol/L solution (in anhydrous dimethyl sulfoxide) added directly to the cell-recording chamber (final concentration 1 μmol/L) for 2–3 min. Excitation was at 488 nm, and emission was collected at >600 nm.

### Statistical Analysis.

Statistical analyses were performed at the level of the cell, and statistics on replicates of *n* individual independent cell experiments from *N* animals are given in the text as *n*/*N*. Data were tested for normality using the Shapiro-Wilk test (Prism7, Graphpad); in any cases where data were skewed, the test was reapplied to log-transformed data. Paired or unpaired *t* tests were performed on normally and log-normally distributed data. Results are presented as mean ± SEM. The limit of statistical confidence was *P* < 0.05.

### Data Availability Statement.

All data and computer codes are available upon request from the authors.

## Supplementary Material

Supplementary File
