# Supplementary material for: Arrhythmogenic late Ca2+ sparks in failing heart cells and their control by action potential configuration
Source: Proc Natl Acad Sci U S A. 2020 Jan 22;117(5):2687–92. doi: 10.1073/pnas.1918649117 (PMC7007549; doi:10.1073/pnas.1918649117)
Supplement: Supplementary File [file pnas.1918649117.sapp.pdf]

Supporting Information for: " Arrhythmogenic late Ca^{2+} sparks in failing heart cells and their control by action potential configuration".

Supplementary Methods

Echocardiography. The echocardiographic examination was performed without sedation using an ultrasound unit (Vivid iQ, GE Healthcare, Little Chalfont, Buckinghamshire, UK) equipped with a 12 MHz phased array transducer. Left ventricular end-systolic (defined as the last frame prior to mitral valve opening) and end-diastolic (defined as the first frame after mitral valve closure) volumes were calculated from the right parasternal long axis four-chamber view, using Simpson's method of discs. The LV area was measured by tracing the endocardial border on each selected image, and maximal left ventricular length was measured from the middle of a line connecting the two mitral valve annuli to the endocardial border of the left ventricular apex. The end-systolic (ESV) and end-diastolic (EDV) volumes were then automatically calculated by the ultrasound machine. The ejection fraction was calculated as ((EDV-ESV)/EDV) x 100.

Isolation of Zebrafish myocytes. Zebrafish were killed by overdose of tricaine (0.05 %) in water. To obtain enough myocytes for experiments, four zebrafish hearts were pooled for enzymatic digestion. The ventricles were dissected and minced in isolation solution containing, in mmol/L: 100 NaCl, 10 KCl, 1.2 KH_2PO_4, 4 $MgSO_4$, 50 taurine, 20 glucose, 10 HEPES, collagenase type I (1.96 mg/ml), trypsin I (0.78 mg/ml) and bovine serum albumin (0.75 mg/ml), pH 6.9 with NaOH. The tissue was gently agitated for 30-40 min, then cells were centrifuged to a pellet and resuspended in isolation solution without enzymes. Experiments were performed in physiological saline (NT) containing, in mmol/L: 150 NaCl, 5.4 KCl, 4 $CaCl_2$, 1.5 $MgSO_4$, 0.4 NaH_2PO_4, 10 HEPES, 10 glucose, pH 7.7. Cells were loaded with cell permeant Fluo-4-AM (Life Technologies Ltd. Paisley UK) (5 μmol/L) for 10 min then placed in a bath on the microscope stage and allowed to de-esterify. Cells were electrically field stimulated with Pt electrodes at 1 Hz at a typical body temperature for zebrafish (27±1 °C) (1). Blebbistatin (Insight Biotechnology Ltd, Wembley UK) (10 μmol/L) was added to inhibit contraction.

I_{Ca} recording. Currents in NT were activated from a holding potential of -80 mV by a ramp (250 ms) and hold at -50 mV for 100 ms (to inactivate the Na^+ current) followed by steps between -50 and +50 mV for 150 ms, before repolarizing to the holding potential. The protocol was performed at 0.2 Hz. I_{Ca} amplitude was measured as the difference between the peak inward current and the current at the end of the step. I_{Ca} was normalized to cell capacitance to calculate I_{Ca} density (pA/pF).

www.pnas.org/cgi/doi/10.1073/pnas.1918649117

Action Potential Clamp Recording. Action potential (AP) clamp involves applying a selected AP waveform as the voltage command to a cell under voltage clamp. The AP waveform may be recorded from the same cell (in current clamp), a typical AP from the same species, or even modified to probe the role of selected ionic currents. During the applied AP, LTCC open in response to the AP waveform as if it were produced endogenously and therefore the Ca^{2+} transient that is produced reveals the functional consequences of the AP waveform on excitation-contraction coupling and Ca^{2+} release. For example, changing the AP waveform to mimic reduced repolarization rate (2), frequency dependent AP duration shortening (3), and heart failure(4), have all been used previously to reveal the role of AP changes on the Ca^{2+} transient and contraction.

Current Clamp V_m Recording. Cells were electrically stimulated by 2 ms depolarizing current injections at 1.3x threshold at 1 Hz. Threshold current was found by progressively increasing pulse amplitude in 0.2 nA increments until APs were consistently evoked.

Calcium imaging analysis. Non-cell background fluorescence from an area adjacent to the cell was subtracted from recordings. Variations in fluorescence due to dye loading was minimized by normalizing fluorescence (F) to resting fluorescence during a 50 ms quiescent period immediately before stimulation (F_0). The F/F_0 recording was converted into units of [Ca^{2+}] using the self-ratio method:

$$[Ca^{2+}]_i = \frac{KR}{(K/[Ca^{2+}]_{rest}) - R + 1}$$

Where K is the *in vivo* affinity of Fluo-4 for Ca^{2+} (K_d ~1000 nmol/L), R is the self-ratio fluorescence (F/F_0), and $[Ca^{2+}]_{rest}$ is the resting Ca^{2+} concentration (~100 nmol/L) (5).

Low-frequency time-averaged fluorescence in x*t* line scan recordings was subtracted from the F/F_0 recording using a low-pass quadratic Savitsky-Golay filter (window size ~101-151 ms) applied along the *t* dimension, for every point in the *x* dimension. These filter values reduced background fluorescence variation due to the underlying Ca^{2+} transient. LCS were then detected using an optimal filter object detection algorithm implemented in MATLAB as described previously (6).

Ca^{2+} transient latency was defined as the time between the AP upstroke crossing -40 mV and cytosolic [Ca^{2+}] exceeding 5 standard deviations above the resting Ca^{2+} signal. Latency was measured at every point along the scan line and the standard deviation was used as a measure of Ca^{2+} release variability. Mean Ca^{2+} release latency was calculated from the average latency across the cell. Ca^{2+}

transient duration was measured as the full duration at half-maximal fluorescence (time from 50 % rise to 50 % decay of the Ca^{2+} transient).

T-Tubule Imaging and Analysis. The t-tubule system was imaged by labelling the sarcolemma with di-8-ANEPPS from a stock 1 mmol/L solution (in DMSO) added directly to the cell recording chamber (final concentration 1 μmol/L) for 2-3 min. Excitation was at 488 nm and emission collected at >600 nm. 3D stacks of images were deconvolved using a 3D Gaussian point spread function. Images were then converted to binary by thresholding then skeletonized using the MATLAB function bwskel (MATLAB version 2019a, MathWorks, MA). Skeletonized images were then processed using appropriately oriented structured elements to segregate transverse (at 90° to the long axis of the cell) and longitudinal (at 0,45, and 135° to the long axis of the cell) tubule elements. The relative tubule orientation was then quantified as the transverse pixel number normalized by the sum of longitudinal and transverse pixels to account for allow for variation in tubule density.

Simulation of ionic currents and LCS. A rabbit ventricular myocyte computer model which couples Ca^{2+} to V_m (7, 8) was used to calculate the magnitude of membrane currents during experimentally recorded EADs. The model features spatially distributed diffusively coupled Ca^{2+} release units and reproduces LCS type events (9). The model code was modified to clamp V_m and Ca^{2+} to values recorded experimentally during EADs. Cross-correlation was used to establish the strength of association and temporal relationship between ionic currents and V_m during EADs. The experimentally recorded V_m during EADs was cross-correlated with I_{NCX} and I_{Ca} calculated by the model. As both I_{Ca} and I_{NCX} are inward (negative) currents, the first negative peak in the cross-correlogram reveals the phase difference between current and V_m. An offset of 0 ms indicates current and V_m are exactly in phase, whereas the current leading V_m means the increase in current precedes the change in V_m. Conversely, a temporal lag indicates current follows V_m.

Supplement References

1. Lawrence C, Mason T (2012) Zebrafish housing systems: a review of basic operating principles and considerations for design and functionality. *ILAR J* 53(2):179–191.

2. Sah R, Ramirez RJ, Backx PH (2002) Modulation of Ca2+ Release in Cardiac Myocytes by Changes in Repolarization Rate. *Circ Res* 90(2):165–173.

3. Hardy MEL, Pervolaraki E, Bernus O, White E (2018) Dynamic Action Potential Restitution Contributes to Mechanical Restitution in Right Ventricular Myocytes From Pulmonary Hypertensive Rats. *Front Physiol* 9:205.

4. Cooper PJ, Soeller C, Cannell MB (2010) Excitation-contraction coupling in human heart failure examined by action potential clamp in rat cardiac myocytes. *J Mol Cell Cardiol* 49(6):911–917.

5. Cannell MB, Cheng H, Lederer WJ (1994) Spatial non-uniformities in [Ca2+]i during excitation-contraction coupling in cardiac myocytes. *Biophys J* 67(5):1942–1956.

6. Kong CHT, Soeller C, Cannell MB (2008) Increasing Sensitivity of Ca2+ Spark Detection in Noisy Images by Application of a Matched-Filter Object Detection Algorithm☆. *Biophys J* 95(12):6016–6024.

7. Restrepo JG, Weiss JN, Karma A (2008) Calsequestrin-mediated mechanism for cellular calcium transient alternans. *Biophys J* 95(8):3767–3789.

8. Terentyev D, et al. (2014) Hyperphosphorylation of RyRs underlies triggered activity in transgenic rabbit model of LQT2 syndrome. *Circ Res* 115(11):919–928.

9. Zhong M, et al. (2018) NCX-Mediated Subcellular Ca2+ Dynamics Underlying Early Afterdepolarizations in LQT2 Cardiomyocytes. *Biophys J* 115(6):1019–1032.

10. Fowler ED, Kong CHT, Hancox JC, Cannell MB (2018) Late Ca2+Sparks and Ripples During the Systolic Ca2+Transient in Heart Muscle Cells. *Circ Res* 122(3):473–478.

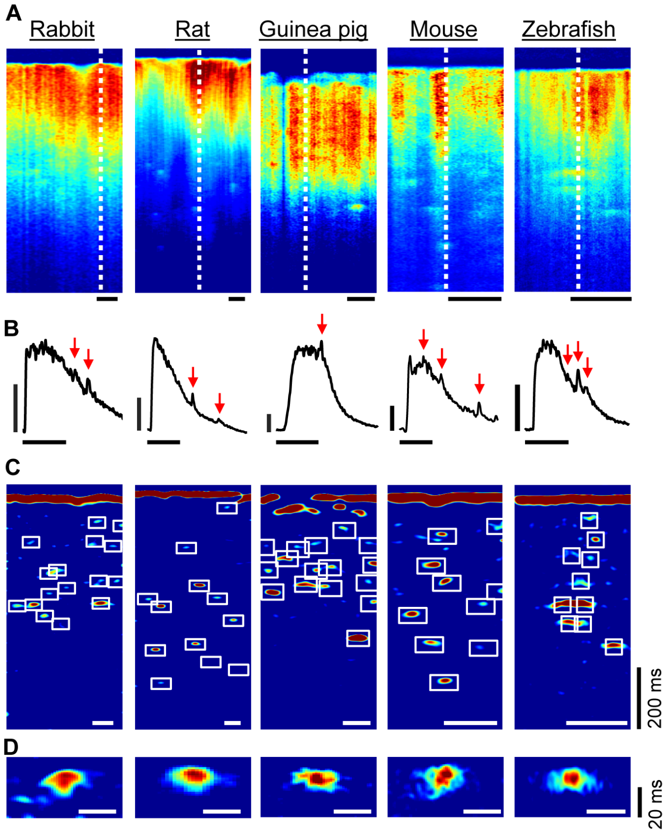

Figure S1. LCS are seen in cardiac cells from a wide variety of species. **A** shows exemplar Ca^{2+} transients from the species indicated. Horizontal scale bars: 5 µm. **B** Profile of the Ca^{2+} transient along the dotted lines shown in **A**. Vertical scale bars: 200 nmol/L. Horizontal scale bars: 200 ms. Red arrows indicate LCS in the declining phase of the transient**. C** Image processing clearly reveals numerous LCS. Horizontal scale bars: 5 µm. **D.** Individual LCS from each species are similar in width and time course. Horizontal scale bars: 2 µm.

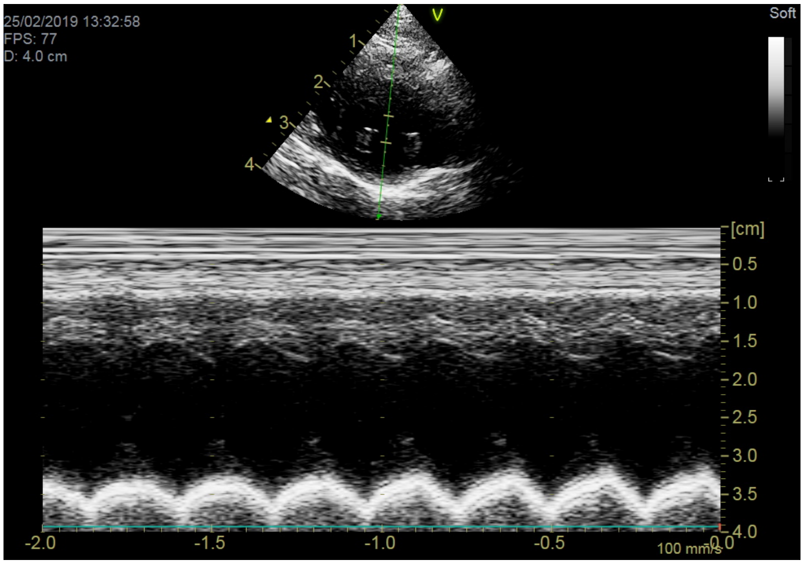

Figure S2. Exemplar M-mode echocardiogram of a post infarct failing (HF) heart with low ejection fraction.

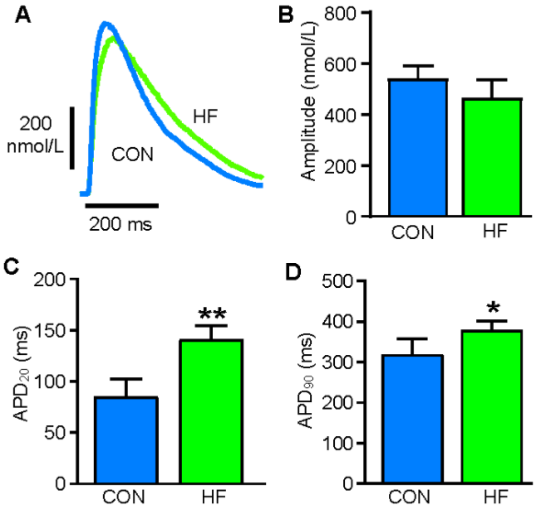

Figure S3. Changes in the Ca^{2+} transient and AP duration in HF. **A**. As might be expected, HF myocyte Ca^{2+} transients had a reduced rate of rise and decline compared to CON. **B**. Although there was a tendency to reduced Ca^{2+} transient amplitude in HF, this was not significant at the group level. **C** The duration of the AP at 20% repolarization was increased in HF cells and **D** at 90% repolarization. *$p<0.05$; ** $p<0.01$, n/N= 16/6 CON ; 14/5 HF.

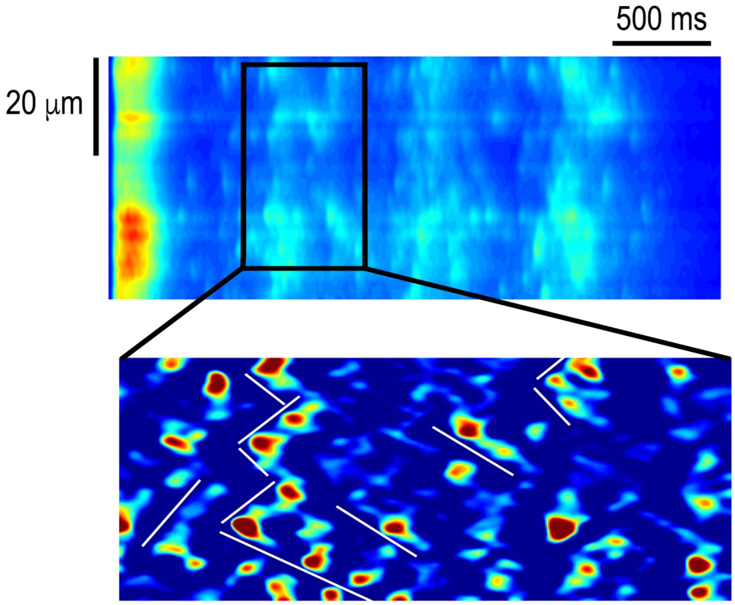

Figure S4 LCS and Ca^{2+} ripples underlying the oscillatory Ca^{2+} release shown in Fig. 2A. Although many LCS can be seen in the upper panels, image processing (lower panel) of the selected region shows numerous chevron-like patterns in underlying LCS activity. Such chevron shapes indicate the presence of Ca^{2+} ripples **(10)** where propagation of CICR via cytoplasmic Ca^{2+} recruits additional LCS sites. This is clarified by the added lines for some of the Ca^{2+} ripples in the lower panel. Note also the very low amplitude chevron shapes that probably reflect Ca^{2+} ripples below the confocal plane.

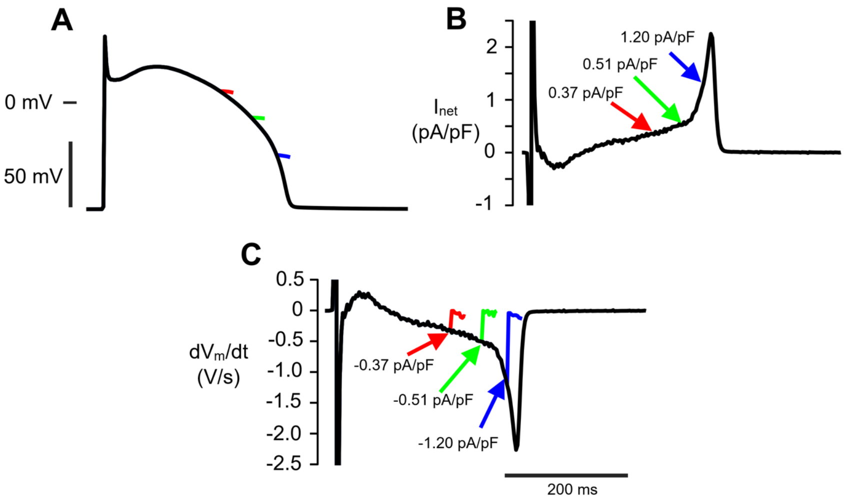

Figure S5. Injection of an Inet balancing current halts AP repolarization. A A model generated A is shown by the black line. B Inet was calculated for the AP in A using the equation Inet= -Cm.dVm/dt (see text). Arrows and associated labels indicate the amplitude of Inet at the indicated points in the AP. C The black line shows the rate of change in membrane potential during the normal AP (dVm/dt). In subsequent simulations, a constant 'balancing' current of equal amplitude but opposite polarity to Inet was injected at the times indicated by arrows. This immediately halted repolarization, as shown by the reduction of dVm/dt to 0 V/s and the cessation of repolarization in the AP (shown by the red, green and blue traces in A).

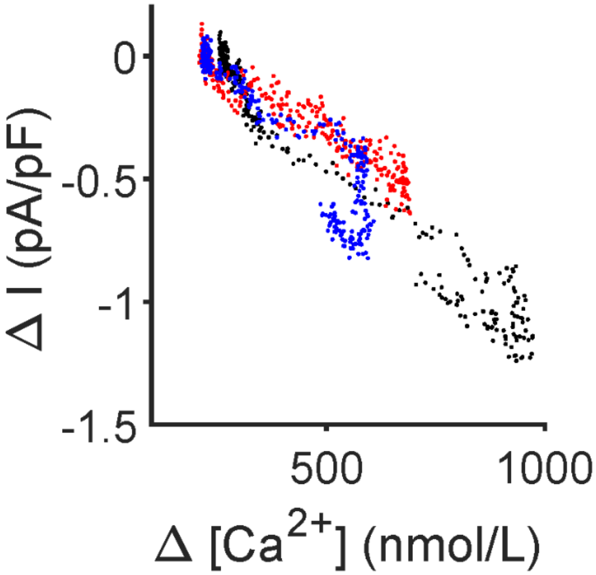

Figure S6. Ca^{2+} dependence of the LCS evoked inward current in Fig. 2. Note the weak voltage dependence of the change in current but strong effect of Ca^{2+} as expected for NCX (*22*). Blue data recorded at V_m = +20 mV; Black at V_m = 0 mV and red at V_m = -20 mV.

Table S1.
Organ weights of CON and HF rabbits. N = 5 CON and 13 HF rabbits. Unpaired t-test.

Parameter	CON			HF			P value
Heart (g)	6.9	±	0.2	12.4	±	0.5	<0.001
Lungs (g)	9.0	±	0.3	12.3	±	0.3	<0.001
Liver (g)	65.3	±	5.1	70.9	±	5.6	0.57
Heart : Tibia (g/cm)	0.73	±	0.02	1.18	±	0.05	<0.001
Lung : Tibia (g/cm)	0.95	±	0.03	1.17	±	0.03	<0.001
Liver : Tibia (g/cm)	6.87	±	0.53	6.70	±	0.50	0.85
